# Shelf sand supply determined by glacial-age sea-level modes, submerged coastlines and wave climate

**DOI:** 10.1038/s41598-019-57049-8

**Published:** 2020-01-16

**Authors:** Marta Ribó, Ian D. Goodwin, Philip O’Brien, Thomas Mortlock

**Affiliations:** 1 The University of Auckland, School of Environment, Auckland, New Zealand; 2 Marine Climate Risk Group, Department of Environmental Sciences, Macquarie University, Sydney, NSW 2109 Australia; 3 UWA Oceans Institute, Crawley, Perth WA, 6009 Australia; 4 Risk Frontiers, St. Leonards, Sydney, NSW 2065 Australia

**Keywords:** Physical oceanography, Palaeoceanography

## Abstract

Submerged paloeshorelines preserved on the continental shelf indicate the depths of the most frequent (modal) low sea-levels within the glacial stages of the Late Quaternary. Here we have determined the south-east Australian shelf configuration when sea level was 40 m and 60 m below present-day sea-level (depths of the most persistent paleoshorelines within the last 120 ka), and we resolve the wave climate variations influencing the sediment transport pathways over this period. We present evidence demonstrating that the combination of shelf morphological evolution, changes in sea-level and variations in wave climate is responsible for latitudinal changes in sediment transport and deposition during the interglacial states. The paleoshoreline and shelf evolution is key to understanding the distribution of present-day shelf sand deposits and the contemporary sand budget response to future wave climate changes.

## Introduction

Long-term coastal evolution is notoriously difficult to resolve due to a poor understanding of the paleo-sediment dynamics involved; especially the changes in sediment transport paths and sediment supply rates, that are related to wave climate and sea-level variability. Coastal sediment dynamics is also influenced by wave climate variations and their impacts upon the shoreline geometry. On stable continental shelves, such as the south east Australia shelf (SEAS)^1–3^, preserved paleoshorelines register the depth at which sea-level persisted for millennia, during which the coastal processes formed depositional and erosional features (e.g., relict barriers, sand spits, shoreface ridges, etc.). Using the records of relative sea-level variations during the Late Quaternary, the most frequent low sea levels can be identified, which define the depth intervals on the shelf (i.e., modal depths) at which paleoshorelines are likely to occur^4^. On wave-dominated coasts, such as the SEAS, the steepness of the shelf is the primary influence on net onshore-offshore sediment budget changes over the long-term^5,6^. Depending on regional variations of shelf morphology and steepness, coastal processes will either be limited to the innermost portion of the shelf (e.g., south of 32°S, where the present-day shelf is relatively narrow and steep (>1°), and dominated by erosional processes) or will cover most of the shelf surface (e.g., north of 32°S, where present-day shelf is shallow and wide, and depositional process are dominant)^7,8^. Additionally, topographic features aligned normal to the coast (i.e., headlands and reefs) also play an important role in determining long-term sedimentation trends, by interrupting the northward drift of sediment at any position of sea-level lower than present.

Together with shelf configuration and margin orientation, modal wave climate is responsible for the long-term delivery of sediment across the shoreface and for the equilibrium shoreline planform orientation^9,10^. Variations in directional wave climate induce changes on the nearshore wave obliquity and alongshore wave energy flux, ultimately influencing the sign and magnitude of sediment transport and deposition^11^.

**Figure 1:**
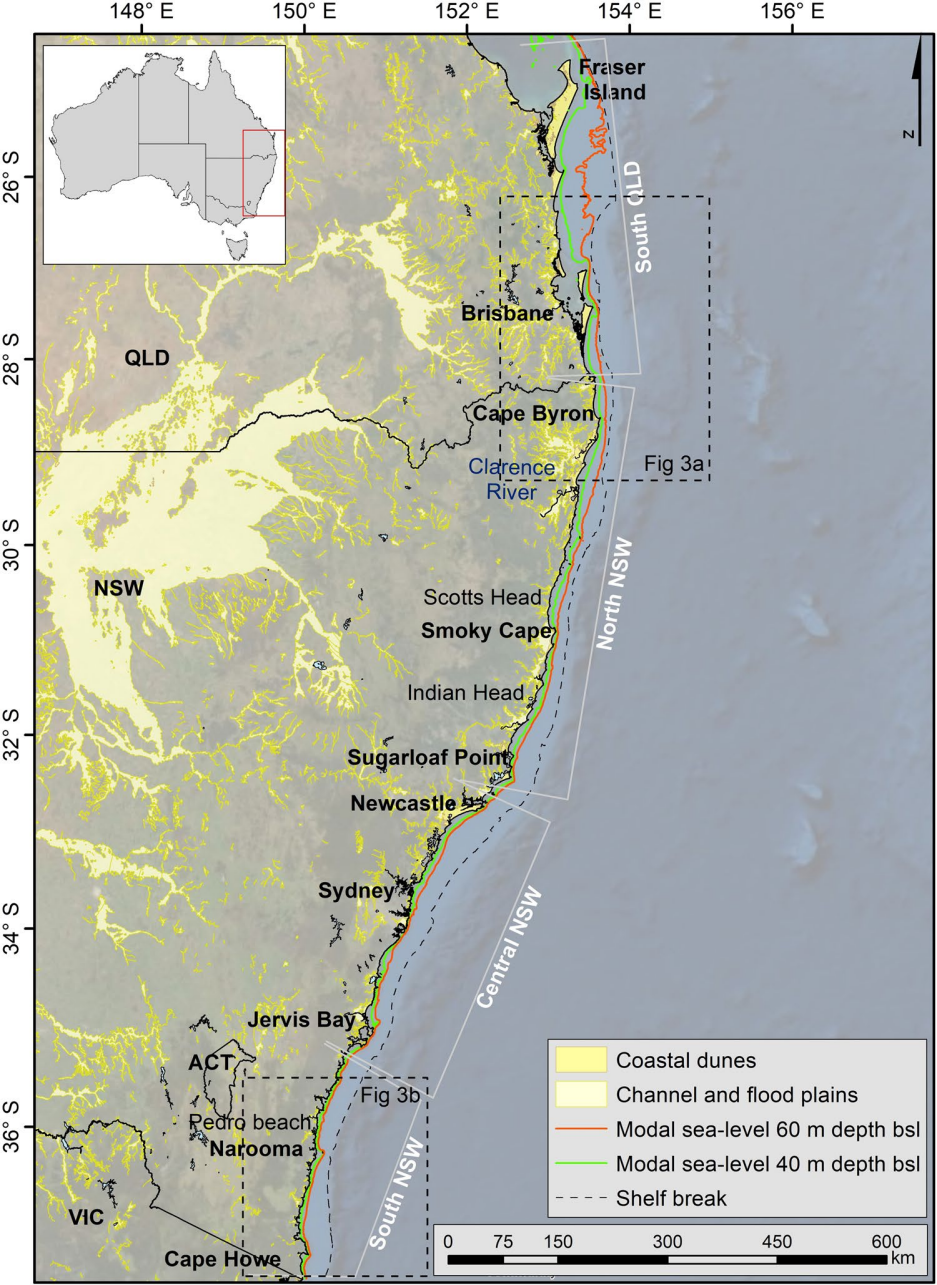
Location map of the south east Australia shelf (SEAS), including the paleoshorelines preserved in the present-day continental shelf, the modal sea levels at 40 m below sea level (bsl) (green) and at 60 m bsl (orange). Dashed line indicates the location of the shelf break (between 120 and 170 m water depth) [map created using software ArcGIS Desktop 10.5.1].

Here we present a comprehensive dataset for characterising SEAS morphology and determining paleoshoreline configuration when sea level was below the modal depth (Fig. 1). We use high-resolution bathymetric data (i.e. single and multibeam sonar, and aerial LiDAR) and legacy data from sediment samples collected at more than 30 sites (early 1980s to present day), to map the grain size distribution and location of reefs and shelf sand deposits along the SEAS (Fig. S1). This information is used together with a hindcast of orbitally forced directional wave climate throughout the glacial cycle, to resolve the connectivity and longshore sediment transport paths during the Late Quaternary glacial and interglacial cycles. The definition of paleo-sediment transport and relict sand bodies is essential data to apply to the projection of modern coastline response to net changes in wave climate, sediment transport and sea levels over the coming century.

## Results and Discussion

### Late-Quaternary sea-level modes

**Figure 2:**
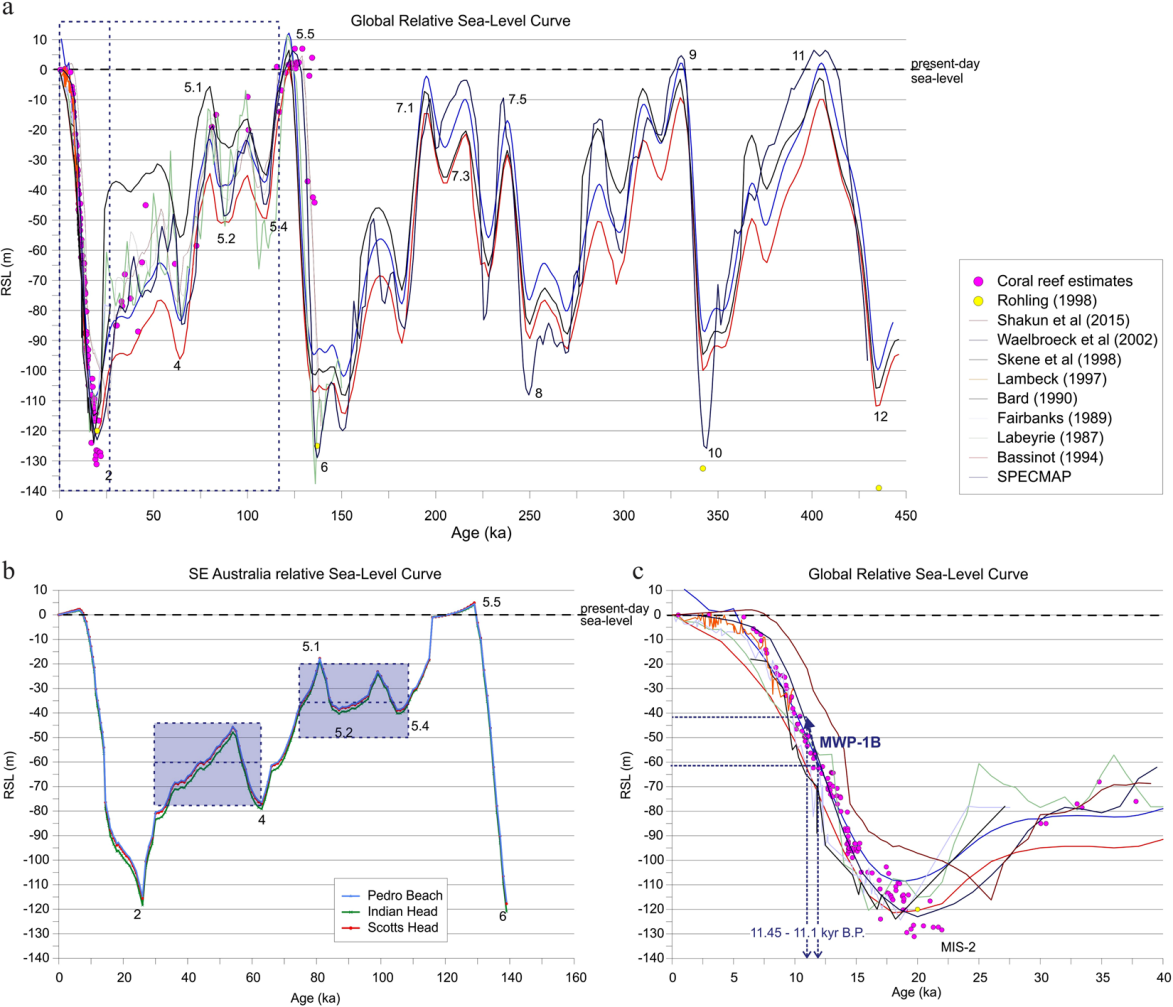
Sea-level curves for the last 400 ka. Bluish shaded areas indicate the Late Quaternary sea-level modes. Numbers indicate the Marine Isotope Stages (MIS), with MIS-2 corresponding to the Last Glacial Maximum (LGM).

Due to the regional variations in shelf morphology, coastal environments along the SEAS are reflective of the magnitude and rate of change of eustatic sea levels. Major glacial and interglacial cycles are identified within the global sea-level reconstructions and are represented by Marine Isotope Stages (MIS), with MIS-2 corresponding to the Last Glacial Maximum (LGM) with a low stand of −120 m below mean sea-level (bMSL) (Fig. 2a)^12–20^. We determined the most frequent low relative sea-levels constituting modal depths within the Late-Quaternary glacio-eustatic sea-level cycle (between MIS-2 and MIS-5, the last interglacial period) using the relative sea-level curve from three locations along the SEAS (i.e., Port Macquarie Coast (Indian Head), Nambucca Coast (Scotts Head), and Moruya Coast (Pedro Beach); see location in Figs. 1 and S1). The first sea-level mode (SLM1) was identified at a depth interval of 30–40 m bMSL, between 110 ka and 80 ka B. P. The second sea-level mode (SLM2) was detected at ~60 m bMSL, between 65 ka and 24 ka B. P. (Fig. 2b). These sea-level modes confirm previous findings^4,21,22^, and indicate the persistent depths at which paleoshorelines were formed along the SEAS and are preserved on the present-day continental shelf (Fig. 1; details in Fig. S2).

At the end of the LGM and during the Post-Glacial Marine Transgression (PGMT) (18 to 10 ka), meltwater from retreating ice sheets increased eustatic sea level, influencing thermohaline circulation dynamics^23^. During this deglaciation, rapid rises in sea-level occurred during meltwater pulse (MWP) 1 A, with a 20 m rise over ~500 years, from ~14.2 to ~13.7 ka B. P^24^, and the MWP-1B, with a sea-level rise of 14 ± 2 m between 11.45 and 11.1 ka B. P^25^ (Fig. 2c). The MWP-1B event commenced when sea-level was at ~60 m bMSL and produced a sea-level transgression at peak rising rates of 40 mm yr^−1^ (Fig. 2c) and produced the most rapid coastal retreat in the past glacial cycle.

### Glacial-age wave climate, longshore sand transport and coastlines

**Figure 3:**
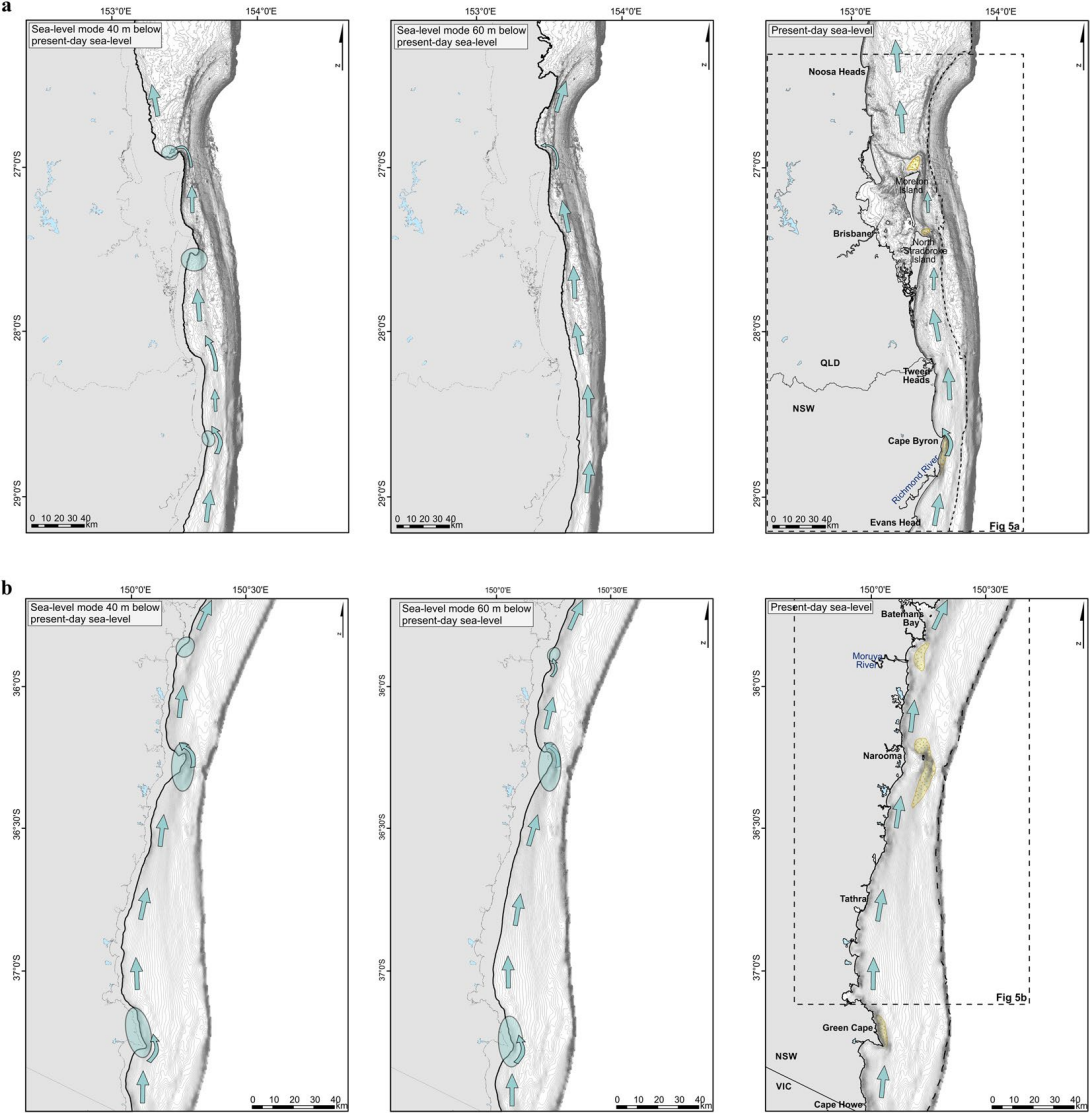
Glacial age (40 m and 60 m sea-level modes) and present-day longshore sediment transport paths, enhanced during dominated SE wave climate (dominated with an MWD of 135° ± Std) at **(a)** south QLD and north NSW and at **(b)** south NSW regions. Circles indicate the areas where sediment transport is most likely to be interrupted, enhancing onshore sand deposition. SSB location is indicated in the present-day shelf configuration [maps created using software ArcGIS Desktop 10.5.1].

The principal climatic factor influencing longshore sand transport connectivity during low sea-level stands is the directional wave climate, changing the wave obliquity with the paleoshorelines and generation of a wave-driven longshore current, inducing realignment of the shoreline geometry, reversal of longshore sand transport, and wave-power modifications to cross-shore sediment transport^26,27^. The SEAS shoreline orientation changes from N-S on the south QLD – north NSW, to a NE-SW orientation on the south NSW region (Fig. S1). Furthermore, the modern modal wave climate along the SEAS is dominated by waves from SSE to ESE^7^ (Fig. S3), which are oriented obliquely to the shoreline, leading to south-to-north alongshore wave energy and littoral transport. This results on a connected northward longshore sand transport and headland bypassing along the NSW shore to north of Fraser Island^28^ (Fig. 3). Storm wave direction has been shown to be the principle driver of headland-bypassing and connectivity of longshore transport^11^. Conversely, shore-normal waves reduce connectivity through minor headland bypassing events or may episodically reverse the net northward transport^29^. Thus, any changes to the directional composition of the wave climate imply changes to the sand transport paths and deposition along the coast.

**Figure 4:**
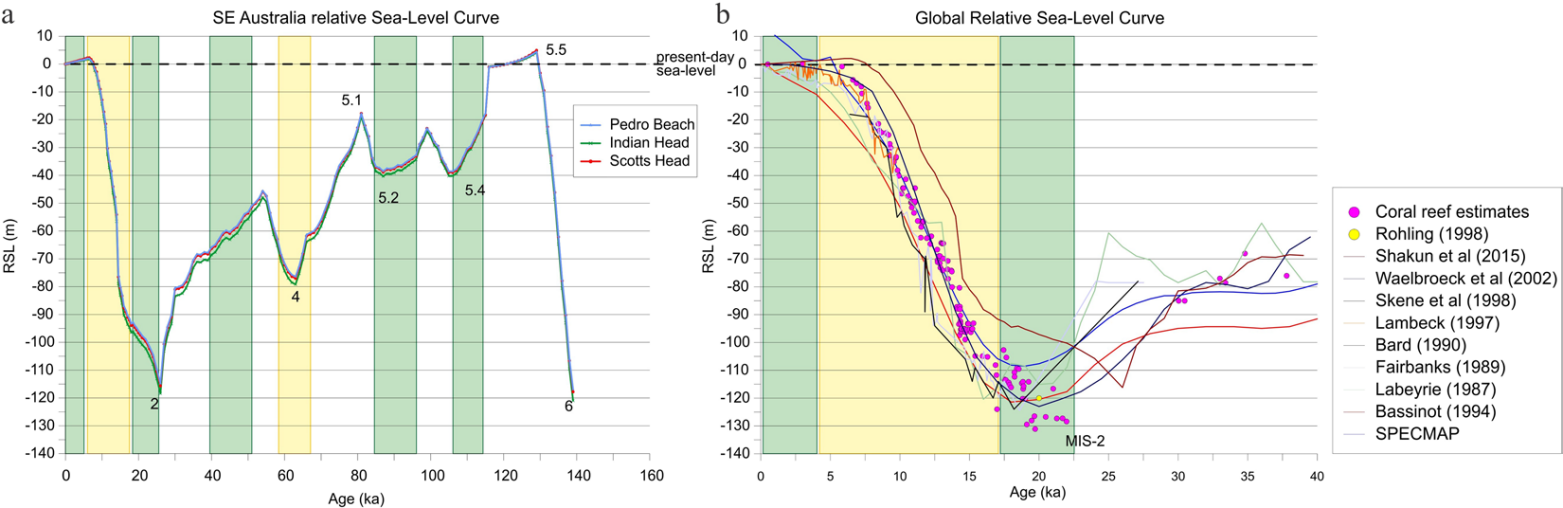
(**a**) SE Australia relative sea-level curve indicating the periods when the longshore transport along the SEAS was active/on (green rectangles) and periods with dominant onshore transport (yellow rectangles). (**b**) SE Australia relative sea-level curve, detail of the last 40 ka. Note that during the MWP-1B (between 11.45–11.1 ka) the longshore transport was off, and sand was predominantly transported on-shore.

Recent work^29^ shows that the variability of the large-scale south-west Pacific atmospheric circulation, the related wind fields and directional wave climate is closely linked to orbital forcing^30^. The combination of orbital obliquity and precession control the strength of seasonality in insolation, the equator-pole temperature gradient and the resultant Tropical extent^31–33^. A strong winter latitudinal temperature gradient (LTG) produces an equatorial shift in the Subtropical Ridge (STR) at 30°S and a resultant directional wave climate along the SEAS that is rotated towards the S^11,30,34^, from mid-latitude westerlies. In contrast a directional wave climate that is rotated towards the E^30^ occurs during a weak winter LTG and a strengthened summer LTG. SLM1 occurs from MIS 5.4 to MIS5.1, and orbitally forced Southerly wave climate peaks at ~110 ka and at 90 ka B. P^30^. Orbitally forced Southerly wave climate also coincides with part of SLM2 during 40–50 ka, B. P. At all other times during SLM1 and SLM2 easterly wave climate dominated. Hence, longshore sand transport dominated SLM1 and onshore sand transport dominated SLM2^30^ (Fig. 4). Both MWP1A and MWP1B occurred during a period of easterly wave climate and weakened longshore sand transport. The implications are that a shore normal wave climate and a rapid sea-level transgression drove an enhanced cross-shelf sand transport mode.

The shelf configuration of paleoshorelines is in agreement with our understanding of orbitally forced wave climate. Shelf configuration during SLM1 revealed that the paleocoast was embayed with many prominent headlands extending into the modal zone at 40 bMSL (Fig. 3). These are identified as locations where updrift headland-attached shelf sediment bodies would have accumulated under an oblique wave climate, similar to the present-day wave climate^35^. We observed that the headland locations during SLM1 coincide with the modern outcrop of relict or ravined sand deposits over the inner shelf (Fig. 3). This indicates that during the SLM1 the deposition of headland-attached shelf sand bodies was ubiquitous along the SEAS. This accords with the dominance of Southerly wave climate and longshore sand transport (Fig. 4). However, during the SLM2, bedrock constraints produced an open coast paleoshoreline, indicating less potential for headland-attached shelf sediment body accumulation, and for a potentially connected longshore sand transport system. We find no evidence for shelf sand bodies, and under an easterly dominant wave climate, we expect that the paleoshorelines forming during SLM2 were characterised by onshore deposition in the form of sand barrier-dune complexes^36,37^, especially on the south QLD - north NSW area^38^, with gentle slopes and smooth and continuous paleoshorline morphology (Fig. 3).

### Distribution of sand deposits over the south east australian shelf

**Figure 5:**
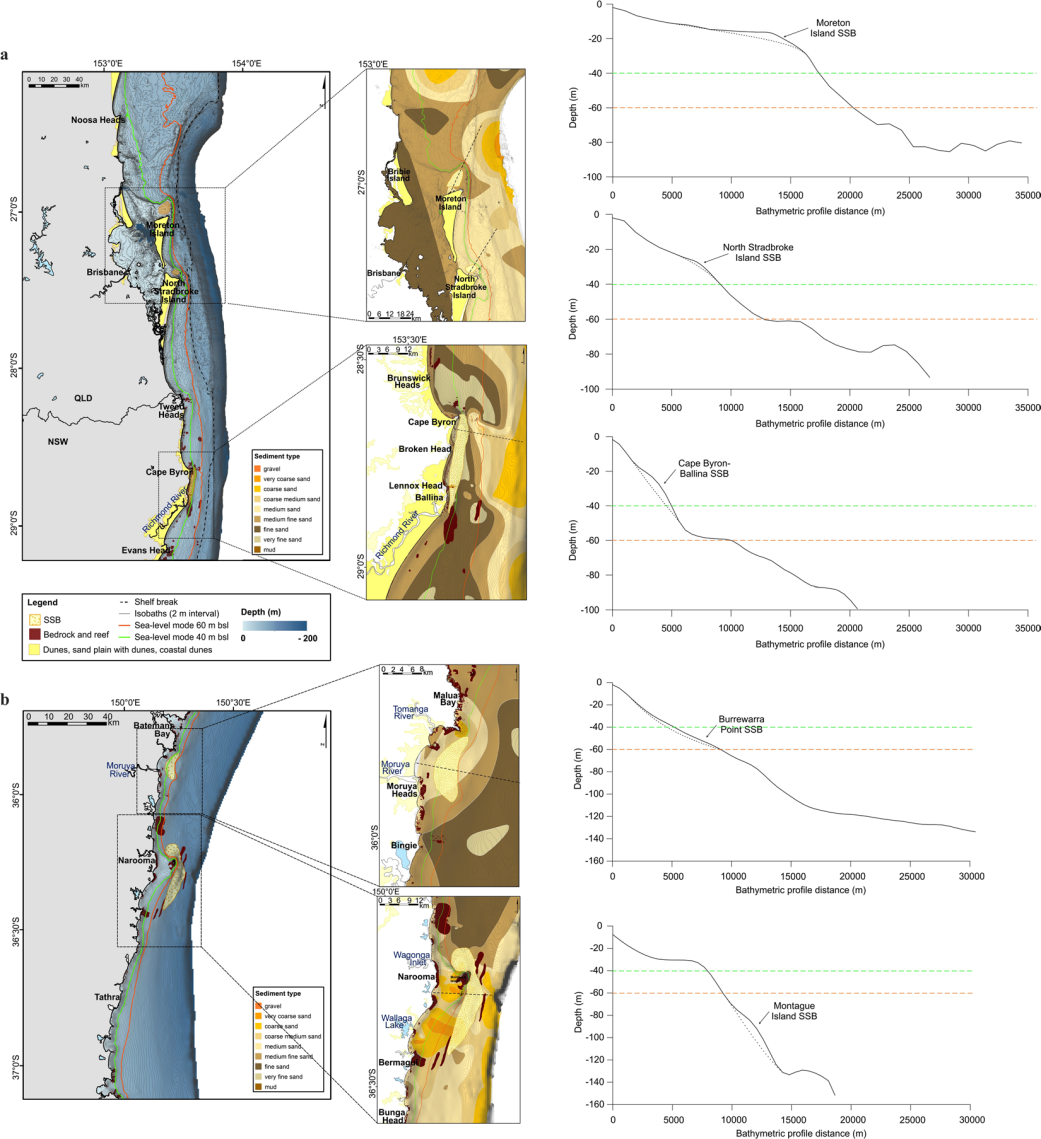
Bathymetric composite map of the **(a)** south QLD and north NSW, and (**b)** south NSW section, including the bedrock, reef and SSBs location and the onshore Quaternary surficial geological units. The 40 m and 60 m isobaths are highlighted in green and orange, respectively, indicating the modal sea-level (i.e., the paleoshorelines location preserved over the present-day south-east Australian shelf). Dashed line squares indicate the zoom-in areas, which include the grain size distribution information and the location of the SSBs. Cross-section bathymetric profiles showed the location of the SSBs, in relation with the modal sea-level depths [map created using software ArcGIS Desktop 10.5.1].

Depositional units on the SEAS are a product of ocean waves and wave-induced currents^39,40^ and their morphology is characterized by a low gradient planar inshore surface (<0.5°–1.5°), with lengths between 5 and 35 km, widths from 1 to 4 km, and thickness from 10 to 50 m^26,40,41^. These shelf deposits are located approximately 2 to 5 km offshore from the modern coast, at depths that vary with latitude along the SEAS, ranging between 10 and 110 m water depth^40,41^. On the northern sections (i.e. south QLD and north NSW, Fig. 1), shelf sand deposits are mainly found in shallower waters (i.e., above the 40 m water depth) than the ones located further south (i.e. central and south NSW, Fig. 1), which are set either in between 40 and 60 m water depths or below the 60 m; however, some exceptions are found (Figs. 5 and S4, S5). These deposition-depth characteristics are in agreement with the wave climate hypothesis, with sand deposited as headland-attached shelf-sand bodies under an enhanced longshore sand transport system during SLM1.

The Cape Byron-Ballina and Montague Island SSBs are the largest sand bodies (with sand volumes of 1.60 km^3^, compared to the average volume of 0.77 km^3^) along the SEAS (Fig. 5). These deposits were both formed from headland sand bypassing, on an oversteepened slope with a component of sand transported offshore from the littoral zone^40,41^. In the Cape Byron area (Fig. 5a), present-day littoral sand supply involves the movement of 300–400,000 m^3^ of sand to the north, that partially contributes to the ongoing deposition of a mid-Holocene SSB sand volume of 1.65 km^3^, with a maximum sand thickness of more than 30 m^21,40^. The majority of the longshore sand transport bypassing Cape Byron continues northward^21^. In contrast, on the south coast the Montague Island sand deposit is located out of the contemporaneous active area of sediment transport and deposition (Fig. 4b), and it contains a similar sand volume (1.60 km^3^) and an even larger maximum sand thickness (up to 48 m) compared to the Cape Byron SSB. This suggest that the longshore sand transport to Montague Island SSB would have been similar to the modern rates observed at Cape Byron^26,27^, and or relates to a deposition time during the glacial, (early SLM2, 55–65 ka) longer than the mid-Holocene to present.

The paleoshoreline and SEAS morphology during the SLM1 was characterized by the presence of headlands, interrupting the longshore sediment transport (Fig. 3). Although the paleoshoreline changed to a smoother and more continuous morphology on the northern section (i.e. south QLD and north NSW, Fig. 1) during SLM2, headlands were still present along the southern SEAS (i.e. central and south NSW, Fig. 1), and sediment transport continued to be interrupted. Therefore, relict shelf sand deposits over the SEAS were formed by waves and wave-induced currents under conditions of falling sea levels and preserved throughout glacial sea level modes, before ravinement during the PGMT^27,36^.

### Shelf morphology and antecedent paleoshoreline recession during MWP1B as a fundamental control of the contemporary sand budget

**Figure 6:**
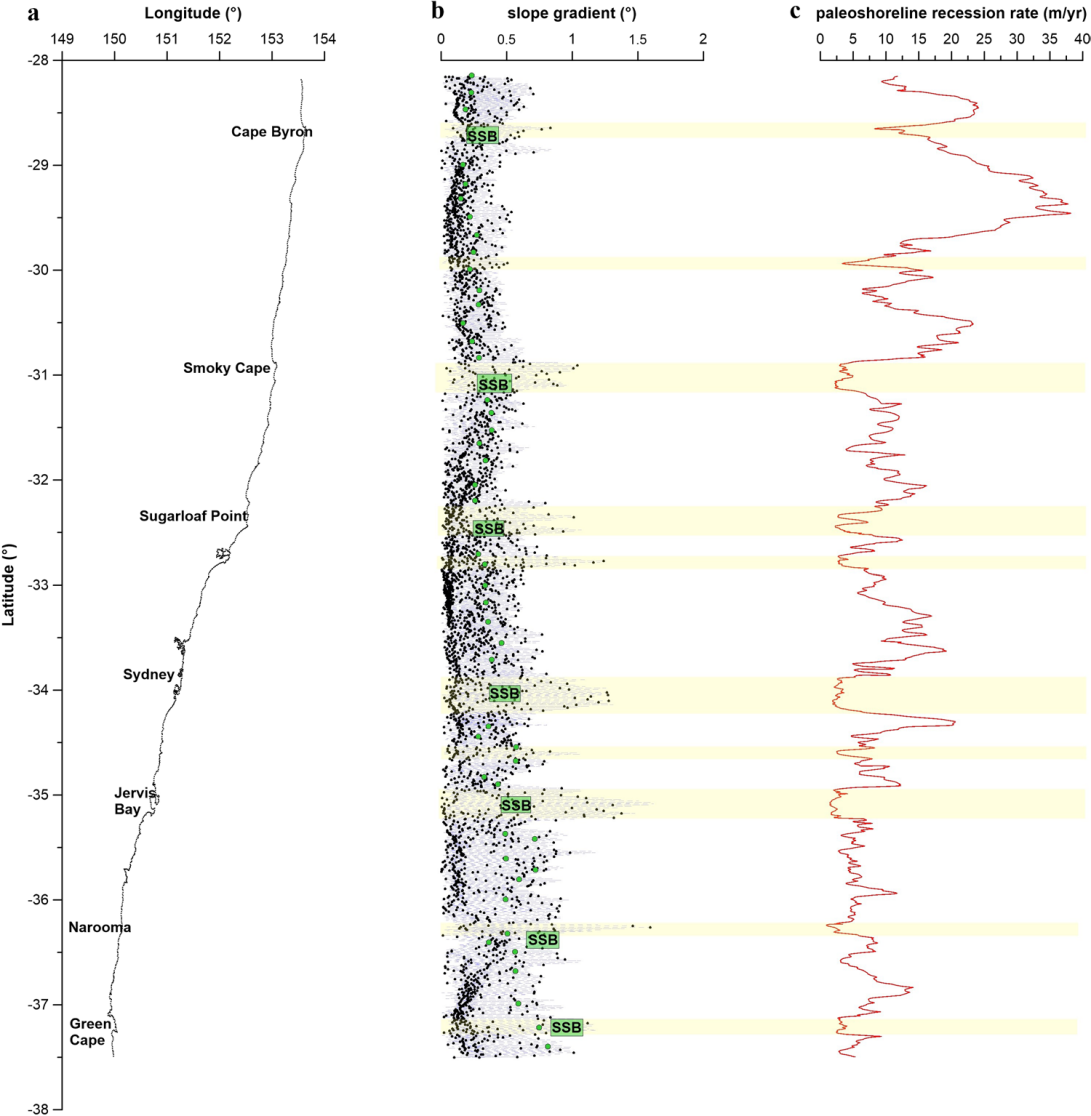
Correlation between the (**a)** coastline morphology and orientation, **(b)** shelf slope gradient (°) along the south-east Australian shelf, and **(c)** the calculated paleoshoreline recession rate (m/yr) during the sea-level rise enhanced by the MWP-1B (11.45–11.1 kya B.P). Yellow bars highlight the areas with the smallest paleoshoreline recession rates coincident with highest slope gradients of ≥1°. Green dots indicate inner shelf slope, and location of the SSB is indicated (adapted from Roy (1998)).

Sand transport along the SEAS depends on the strength and net-direction of wave-induced currents at the seabed, which is directly related to the water depth-wave base relationship^39^. Moreover, the amount of sand transported and accumulated along the shelf is influenced by the slope gradient of the inner shelf surface^28^. Our findings demonstrate a direct relation between the location of the shelf sand deposits along the SEAS, the inner shelf slope gradient, and the paleoshoreline recession rate during the rapid sea-level rise through the MWP-1B (Fig. 6). The maximum rates of sea-level rise approach the 40 mm yr^−1^ for the period of approx. 350 yr^26^, with a paleoshoreline recession rate ranging between 0.9 and 38 m yr^−1^, varying with latitude due to the changes in slope gradient (Fig. 6). Implicit is the assumption that the paleoshore recession kept pace with sea-level rise.

On the south QLD - north NSW coast, the shelf presents gentle slopes and high paleoshoreline recession rates are interpreted (avg. 10 m yr^−1^; maximum of 38 m yr^−1^). Conversely, recession rates decrease towards the south NSW region (avg. 5 m yr^−1^), where the shelf slope becomes steeper. Thus, the slowest paleoshoreline recession rate occurs where inner shelf slope gradient is steepest (≥1°), which also coincides with the areas where the shelf sand bodies are located (Fig. 6).

As previous studies have shown, coastal recession does not conform to the Bruun Rule under scenarios of extreme sea level rise, being one of the major uncertainties associated with the role played by the slope of the shelf in retreat rates^42–45^. For rapid SLR such as MWP1B, the shoreline translation rate on the flat shelf in south QLD - north NSW of max. 38 m/yr is 10 times the rate predicted from the Bruun Rule (R = 100 × the SLR)^44,45^. Whereas the shoreline translation rate on the steep southern NSW shelf south NSW coast of 5 m/yr is in accordance with the Bruun Rule. This indicates the shoreface slope threshold for reliable application of the Bruun Rule, and that on flatter shoreface slopes, inundation and overstepping processes dominate the coastal evolution^46^. Shallow shelves are expected to exert a larger sediment demand than steeper shelves, resulting in increased rates of shoreface recession in order to maintain the equilibrium under rapid SLR^47^.

**Figure 7:**
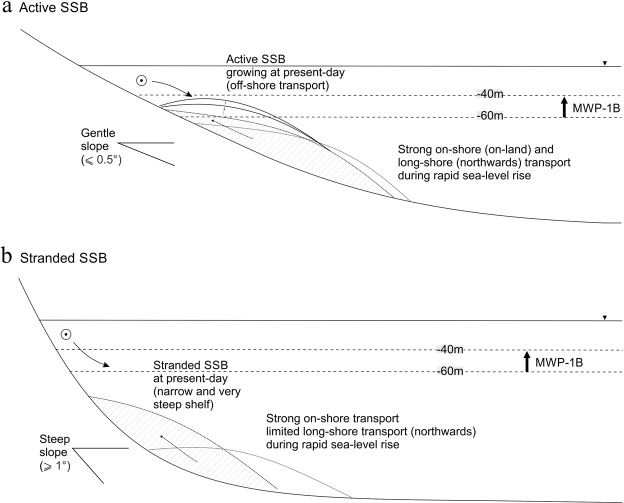
Conceptual model showing the shelf sand deposits evolution with changes in sea-level over shallow **(a)** and steep **(b)** slope gradients (oblique wave energy is assumed for both scenarios).

The 40–50 ka period of SLM2 at close to 40 m depth BSML is the main period where a southerly wave domination had the potential to transport sand alongshore open compartments from northern NSW into southern QLD (Fig. 4). This led to reworking of sediments on the shelf (i.e., absence of large fluvial supply of new sediments onto the shelf during the last cycle^41^, and high content of very mature quarts and feldspar grains in the sediment, indicate reworking in the marine system during the Quaternary^26^), mainly occurring on the northern SEAS (i.e. south QLD and north NSW, Fig. 1), but also in any area where the slope gradient is gentle (≤0.5°), resulting in sand deposits being located at water depths between 10 and ~50 m over the present-day shelf (e.g., Moreton Island SSB and Stradbroke Island SSB (Fig. 5a) and Burrewarra Point SSB (Fig. 5b)). The later shift to easterly wave climate during the PGMT led to the ravinement of these shelf sand bodies and cross-shelf sand transport to nourish the great sand islands (Stradbroke, Moreton and Fraser islands; Fig. 5a), with an onshore accumulation of sands during the early Holocene. The ravined shelf sand bodies are currently located within the active zone of the lower shoreface (i.e., 20 m to 40 m water depth^28^), and potential is the source of modern shoreline sand supply, due to disequilibrium stress with the modern SL highstand. (Fig. 7a). Conversely, on-shore transport was strong during the MWP-1B on the steeper southern NSW shelf. However, due to the steepness of the shelf (≥1°), the cross-shelf sand movement was restricted, and the shelf sand deposits became stranded, being preserved at water depths ≥60 m (out of the active wave base zone) over the present-day shelf (Fig. 7b).

## Conclusions

Current and future sea-level rise has been a catalyst for regional assessment of coastal vulnerability in Australia and elsewhere around the world. At the core of these assessments has been an attempt to determine the discrete coastal compartmentalisation where sediment exchange can be determined as a closed budget. A negative sediment budget exposes a coast to increased vulnerability to SLR and rapid coastal recession. Conversely, a positive sediment budget may produce a negative feedback to SLR and enable a shoreline to maintain its geographical location despite moderate rates of SLR (~10 mm/yr). Most methods of delineating coastal compartments are determined from the configuration of headlands, reefs and the enclosure of the upper shoreface. Our results suggest that the antecedent glacial-age coastlines, wave climate and sediment transport and shelf sand-deposition on the now drowned shelves are key to determining the modern sediment budget. In the case of eastern Australia, the lagged sand supply throughout the Holocene to present is due to the onshore sediment transport during the PGMT (i.e., sediment from ravined coasts being transported from the shoreface to the shoreline).

We recommend that future coastal compartmentalisation is based on the integrated modern and glacial-age shorelines and shoreface sediment deposits (i.e. revision of current coastal compartments delineation, considering no only the location of headlands or major river entrances, but also the location of SSB, which is influenced by the glacial age sand supply along and cross-shore, and affecting the present-day exchange sand between compartments).

Our results indicate that wave climate variability in the subtropical to mid-latitudes is far more variable than previously thought. The orbital control on wave climate variability provides a new benchmark to assess projections of future wave climate change using Global Climate Models (GCM’s). The assumption that the modal wave climate for the past half century is the best baseline for predicting future coastal change is flawed. Our wholistic approach to studying the glacial to interglacial coastal evolution provides a strong framework for identifying relict sediment deposits or lagged and ongoing sediment supply from shelves in disequilibrium stress and assessing the potential for future coastal change.

## Methods

### Data compilation

The data used in this study originates from a wide range of sources such as the Marine Sediment (MARS) database; Geoscience Australia; OzCoast database, NSW Public Works Department, NSW Department of Mineral Resources-Geological Survey; NSW Department of National Development; NSW Office of Environment and Heritage (OEH); NSW Marine Parks Authority; Geological Survey of NSW; University of Sydney and Macquarie University data holdings.

Information on sediment type and grain size was obtained from sediment samples, and used to build a complete grain size distribution map over the South-east Australian shelf. This map also includes the location of shelf sand deposits and nearshore and inner shelf reefs and bedrock-reefs.

Seafloor geomorphology was interpreted from bathymetric data, which integrate airborne LiDAR (Light Detection and Ranging), multibeam and single beam echosounder data.

Information of the Onshore Quaternary surficial geological units was extracted from The Surface Geology of Australia 1:1 M scale dataset^48^.

### Figures

Maps presented in Figs. 1, 3 and 5 and Supplementary Material were created using software ArcGIS Desktop 10.5.1 (https://www.esri.com/).

## Supplementary information

Supplementary Information.

## References

[1] Ollier CD (1982). The great escarpment of eastern Australia: tectonic and geomorphic significance. Jour. Geol. Soc. Australia.

[2] Stagg HMJ (1999). Architecture and evolution of the Australian continental margin. AGSO Journal of Australian Geology & Geophysics.

[3] Pain, C. F., Pillans, B. J., Roach, I. C., Worrall, L. & Wilford, J. R. Old, flat and red – Australia’s distinctive landscape. In: Blewett, R. (Ed.), Shaping a Continent, Building A Nation: A Geology of Australia. *Geoscience Australia and ANU E Press, Canberra*, 225–275 (2012).

[4] Brooke BP, Nichol SL, Huang Z, Beaman RJ (2017). Palaeoshorelines on the Australian continental shelf: Morphology, sea-level relationship and applications to environmental management and archaeology. Continental Shelf Research.

[5] Roy, P. S., Cowell, P. J., Ferland, M. A. & Thom, B. G. Chapter 4: Wave dominated coasts. In: Carter, R. W. G. and Woodroffe, C. D. (eds), Coastal Evolution, Cambridge University Press, 121–186 (1994).

[6] Cowell PJ, Roy PS, Jones RA (1995). Simulation of large-scale coastal change using a morphological behaviour model. Marine Geology.

[7] Roy PS, Thom BG (1981). Late Quaternary marine deposition in New South Wales and southern Queensland – an evolutionary model. Journal of the Geological Society of Australia.

[8] Thom BG, Keene JB, Cowell PJ, Daley M (2010). East Australian marine abrasion surface. Geological Society, London, Special Publications.

[9] Mortlock TR, Goodwin ID (2016). Impacts of enhanced central Pacific ENSO on wave climate and headland-bay beach morphology. Continental Shelf Research.

[10] Ranasinghe R, McLoughlin R, Short A, Symonds G (2004). The Southern Oscillation Index, wave climate, and beach rotation. Marine Geology.

[11] Goodwin ID, Mortlock TR, Browning S (2016). Tropical and extratropical-origin storm wave types and their influence on the East Australian longshore sand transport system under a changing climate. Journal of Geophysical Research: Ocean.

[12] Shakun JD, Lea DW, Lisiecki LE, Raymo ME (2015). An 800-kyr record of global surface ocean δ18O and implications for ice volume-temperature coupling. Earth and Planetary Science Letters.

[13] Waelbroeck C (2002). Sea-level and deep water temperature changes derived from benthic foraminifera isotopic records. Quaternary Science Reviews.

[14] Skene KI, Piper DJW, Aksu AE, Syvitski JPM (1998). Evaluation of the global Oxygen Isotope Curve as a proxy for the Quaternary Sea Level by modeling of Delta Progradation. Journal of Sedimentary Research, section B: Stratigraphy and Global studies.

[15] Lambeck K (1997). Sea-level change along the French Atlantic and Channel coasts since the time of the Last Glacial Maximum. Palaeogeogr. Palaeoclimatol. Palaeoecol..

[16] Bard E, Hamelin B, Fairbanks RG (1990). U-Th ages obtained by mass spectrometry in corals from Barbados: sea level during the past 130,000 years. Nature.

[17] Fairbanks RG (1989). A 17,000-year glacio-eustatic sea level record: influence of glacial melting rates on the Younger Dryas event and deep-ocean circulation. Nature.

[18] Labeyrie LD, Duplessy JC, Blanc PL (1987). Variations in mode of formation and temperature of oceanic deep waters over the past 125,000 years. Nature.

[19] Bassinot FC (1994). The astronomical theory of climate and the age of the Brunhes-Matuyama magnetic reversal. Earth and Planetary Science Letters.

[20] Bassinot, F. C. SPECMAP. In: Gornitz V. (eds) Encyclopedia of Paleoclimatology and Ancient Environments. Encyclopedia of Earth Sciences Series. Springer, Dordrecht (2009).

[21] Harris PT (2005). Tidally-incised valleys on tropical carbonate shelves: an example from the northern Great Barrier Reef, Australia. Marine Geology.

[22] Harris, P. T. & MacMillan-Lawler, M. Global Overview of Continental Shelf Geomorphology Based on the SRTM30_PLUS 30-Arc Second Database. In: Finkl, C.W., Makowski, C. (Eds.), Seafloor Mapping along Continental Shelves. Springer International Publishing Switzerland, 169–190 (2016).

[23] Weaver AJ, Saenko OA, Clark PU, Mitrovica JX (2003). Meltwater Pulse 1A from Antarctica as a Trigger of the Bølling-Allerød Warm Interval. Science.

[24] Clark PU, Mitrovica JX, Milne GA, Tamisiea ME (2002). Sea-Level Fingerprinting as a Direct Test for the Source of Global Meltwater. Pulse 1A. Science.

[25] Abdul NA, Mortlock RA, Wright JD, Fairbanks RG (2016). Younger Dryas sea level and meltwater pulse 1B recorded in Barbados reef crest coral Acropora palmata. Paleoceanography.

[26] Roy, P. S. Sand Deposits of the NSW Inner Continental Shelf. *Geoscience Survey Report* (2001).

[27] Field ME, Roy PS (1894). Offshore transport and sand-body formation: evidence from a steep, high-energy shoreface, southeastern Australia. Journal of Sedimentary Petrology.

[28] Boyd R, Ruming K, Goodwin I, Sandstrom M, Schröder-Adams C (2008). Highstand transport of coastal sand to the deep ocean: A case study from Fraser Island, southeast Australia. Geology.

[29] Goodwin ID, Freeman R, Blackmore K (2013). An insight into headland sand bypassing and wave climate variability from shoreface bathymetric change at Byron Bay, New South Wales, Australia. Marine Geology.

[30] Goodwin, I. D. Orbital forcing of subtropical wind and wave climate (in prep., 2019).

[31] Hays JD, Imbrie J, Shackleton NJ (1976). Variations in the Earth’s Orbit: Pacemaker of the Ice Ages. Science.

[32] Beck, H. E. *et al*. Present and future Köppen-Geiger climate classification maps at 1-km resolution. *Scientific Data*, vol. 5, Article number: 180214 (2018). 10.1038/sdata.2018.214 PMC6207062 30375988

[33] Davis BAS, Brewer S (2009). Orbital forcing and role of the latitudinal insolation/temperature gradient. Climate Dynamics.

[34] Mortlock TR, Goodwin ID (2015). Directional wave climate and power variability along the Southeast Australian shelf. Continental Shelf Research..

[35] Goodwin, I. D., Ribo, M. & Mortlock, T. Coastal sediment compartments, wave climate and centennial-scale sediment budget: The south-eastern Australian example. In Jackson, D. and Short, A., (eds.) Sandy Beach Morphodynamics. Chapter 25. Elsevier. (2019).

[36] Thom BG, Bowman GM, Roy PS (1981). Late Quaternary Evolution of Coastal Sand Barriers, Port Stephens – Myall Lakes area, central New South Wales, Australia. Quaternary Research.

[37] Thom B, Hesp P, Bryant E (1994). Last glacial “coastal” dunes in Eastern Australia and implications for landscape stability during the Last Glacial Maximum. Palaeogeography, Palaeoclimatology, Palaeoecology.

[38] Booke BP, Pietsch TJ, Olley JM, Sloss CR, Cox ME (2015). A preliminary OSL chronology for coastal dunes on Moreton island, Queensland, Australia – Marginal deposits of a large-scale quaternary shelf sediment system. Continental Shelf Research.

[39] Roy, P. S. Chapter 25, Cainozoic geology of the New South Wales coast and shelf. In Geology of New South Wales—Synthesis. Volume 2, Geological Evolution: Precambrian to Present (Scheikner, E. & Basden, H., eds.). Geological Survey of New South Wales, Australia. Memoirs, Geology 13, pp. 361–385 (1998).

[40] Roy, P. S. Inner Continental Shelf Sand Deposits: SE Australia. University of Sydney Institute of Marine Science & School of Geosciences. *Internal Report*., pp. 170 (2006).

[41] Ferland, M. A. Shelf Sand Bodies in South Eastern Australia. PhD Thesis, Department of Geography. The University of Sydney, p. 183 (1990).

[42] Bruun P (1962). Sea-Level Rise as a Cause of Shore Erosion. American Society of Civil Engineers Journal of the Waterways and Harbours Division..

[43] Ranasinghe R, Callaghan D, Stive MJF (2012). Estimating coastal recession due to sea level rise: beyond the Bruun rule. Climatic Change.

[44] Cooper JAG, Pilkey OH (2004). Sea-level rise and shoreline retreat: time to abandon the Bruun Rule. Global and Planetary Change.

[45] Cowell PJ, Roy PS, Jones RA (1995). Simulation of large-scale coastal change using a morphological behaviour model. Marine Geology.

[46] Kinsela, M. A. Shoreface Response to Sea Level Change and the Evolution of Barrier Coasts. PhD thesis. School of Geosciences. The University of Sydney, pp. 367 (2014).

[47] Daley, M. J. A. Disequilibrium-Stress Induced Shoreface Evolution. PhD thesis. School of Geosciences. The University of Sydney, pp. 171 (2011).

[48] Raymond, O. L., Liu, S., Gallagher, R., Highet, L. M. & Zhang, W. Surface Geology of Australia, 1:1 000 000 scale. 2012 edition [Digital Dataset, http://www.ga.gov.au,]. Geoscience Australia, Commonwealth of Australia, Canberra (2012).

