# Supplementary material for: Shelf sand supply determined by glacial-age sea-level modes, submerged coastlines and wave climate
Source: Sci Rep. 2020 Jan 16;10:462. doi: 10.1038/s41598-019-57049-8 (PMC6965639; doi:10.1038/s41598-019-57049-8)
Supplement: Supplementary file 1 — Supplementary Information. [file 41598_2019_57049_MOESM1_ESM.docx]

**Shelf sand supply determined by glacial-age sea-level modes, submerged coastlines and wave climate**

Marta Ribó^*1, 2^, Ian D. Goodwin^2, 3^, Philip O’Brien^2^, Thomas Mortlock^2, 4^

^1^ The University of Auckland, School of Environment, Auckland, New Zealand. ^2^ Marine Climate Risk Group, Department of Environmental Sciences, Macquarie University, Sydney, NSW 2109, Australia. ^3^ UWA Oceans Institute, ^4^ Risk Frontiers, St. Leonards, NSW 2065, Australia.

**Supplementary Information**

**FIGURE S1**


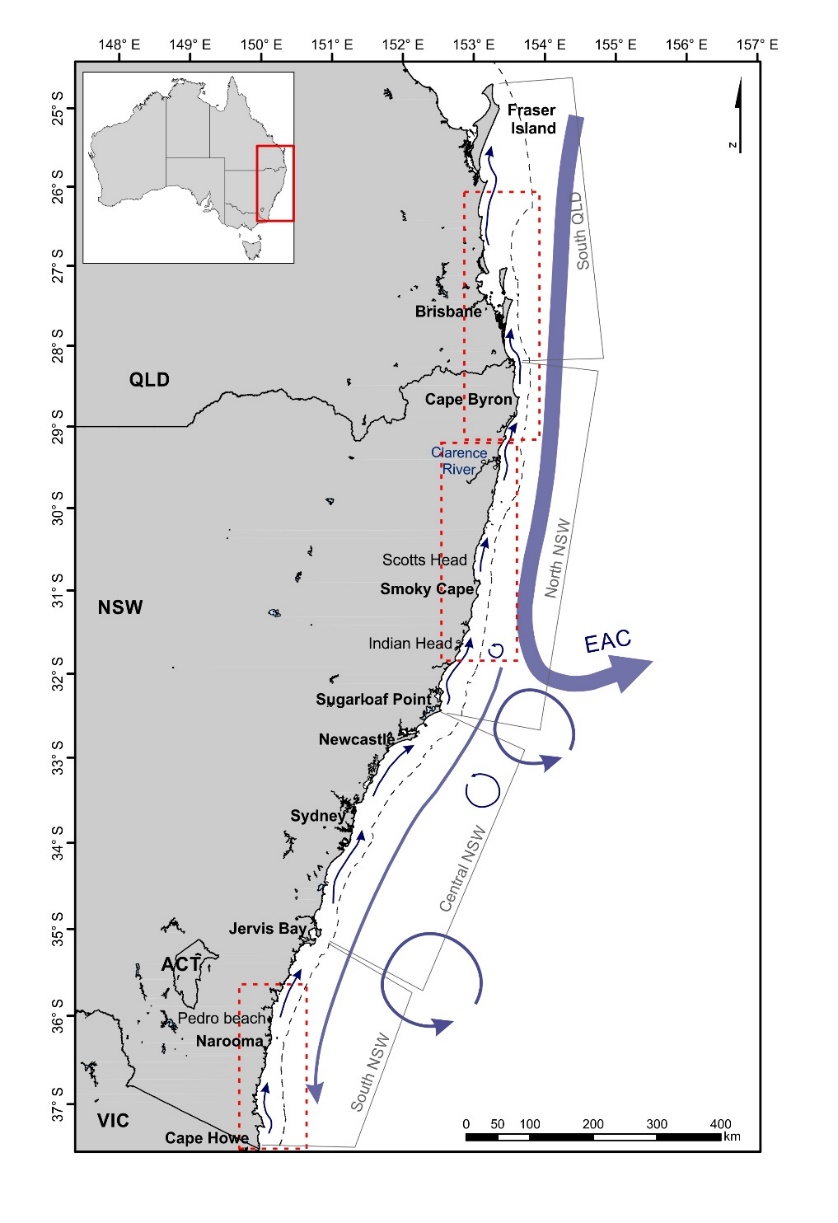

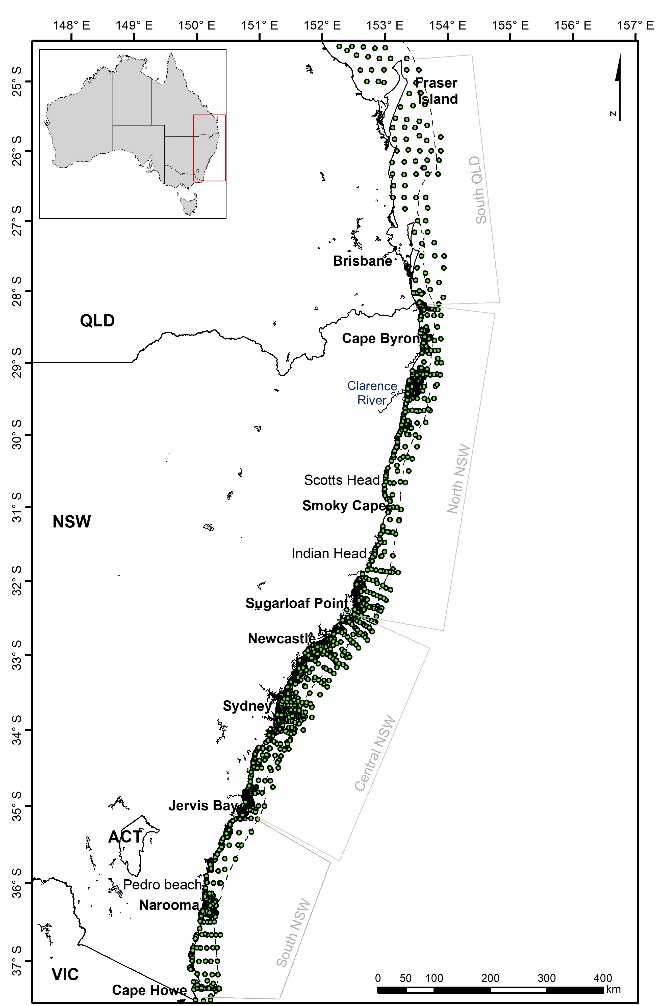

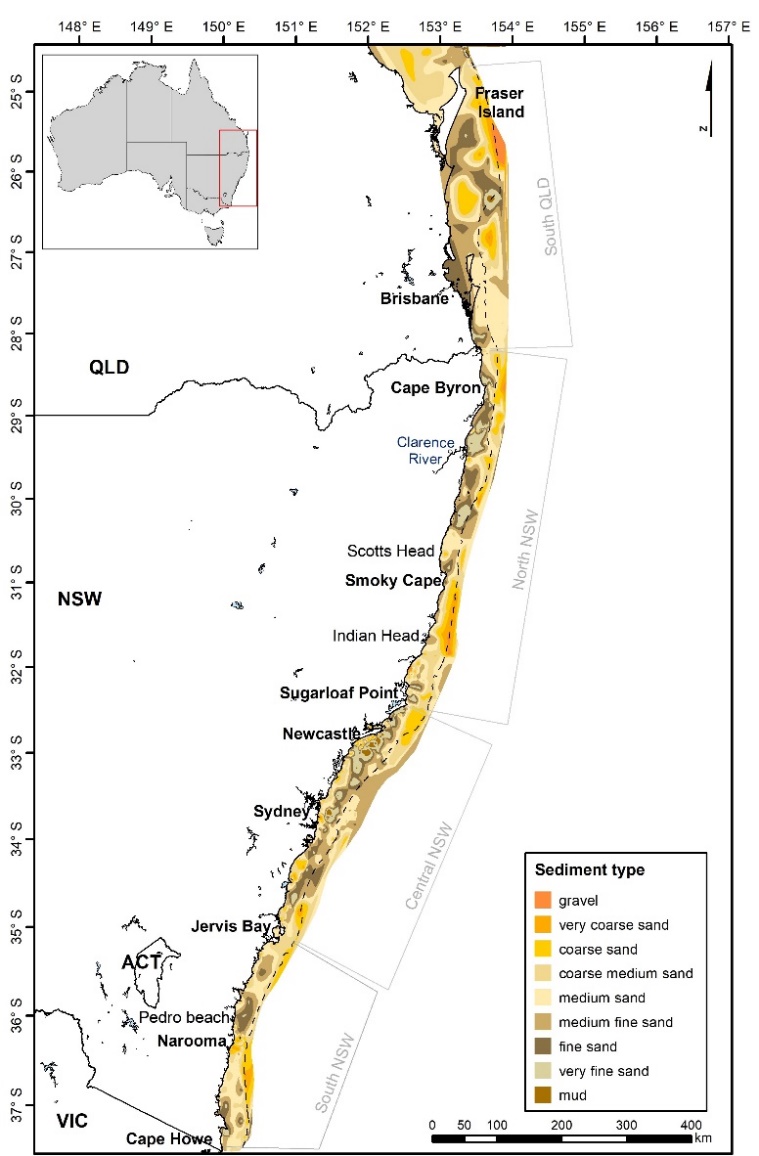


**C**

**B**

**A**

**Figure S1. A)** Location map of the south-eastern Australian margin indicating general circulation. Red dashed boxes indicate the two regions were the wave obliquity is assessed. Wave climate modes main directions are indicated with the blue, red and green filled arrows. **B)** Location of the sediment samples (green dots) with grain size information used in this study. **C)** Revised grain size distribution map of the SEAS created using the compiled information from sediment samples collected along the continental shelf. Dashed line indicates the location of the shelf break (between 130 and 170 m water depth).

**FIGURE S2**

**
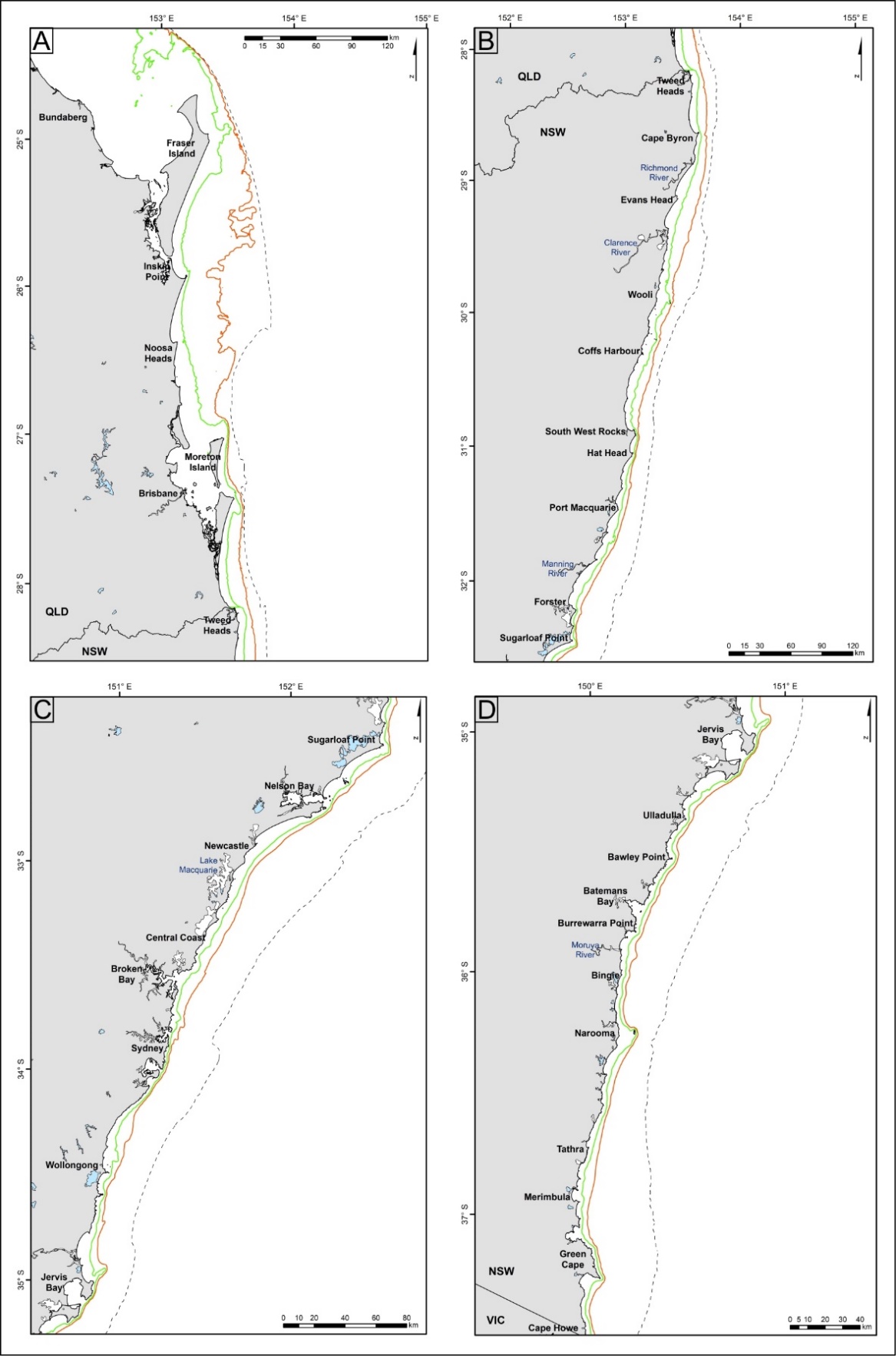
**

**Figure S2.** South-eastern Australian map, separated in (A) South-East Queensland, (B) North NSW, (C) Central NSW and (D) Southern NSW. All Maps include the paleoshorelines preserved in the present-day continental shelf, which follow the 40 m (green) and 60 m (orange) isobaths. Dashed line indicates the location of the shelf break (between 120 and 170 m water depth).

On the south and central NSW coast, the paleoshoreline depths are restricted to the innermost section of the modern inner shelf (Fig. S3). From ~32° S northwards, the paleoshorelines expand seawards covering most of the shelf of north NSW and south QLD. This variation in modal sea-level position coincides with the latitudinal changes of shelf width and morphology (note that the shelf break is observed at ~120 m water depth in the north and deepens towards the south, where the shelf break is at ~165 m water depth).

**FIGURE S3**


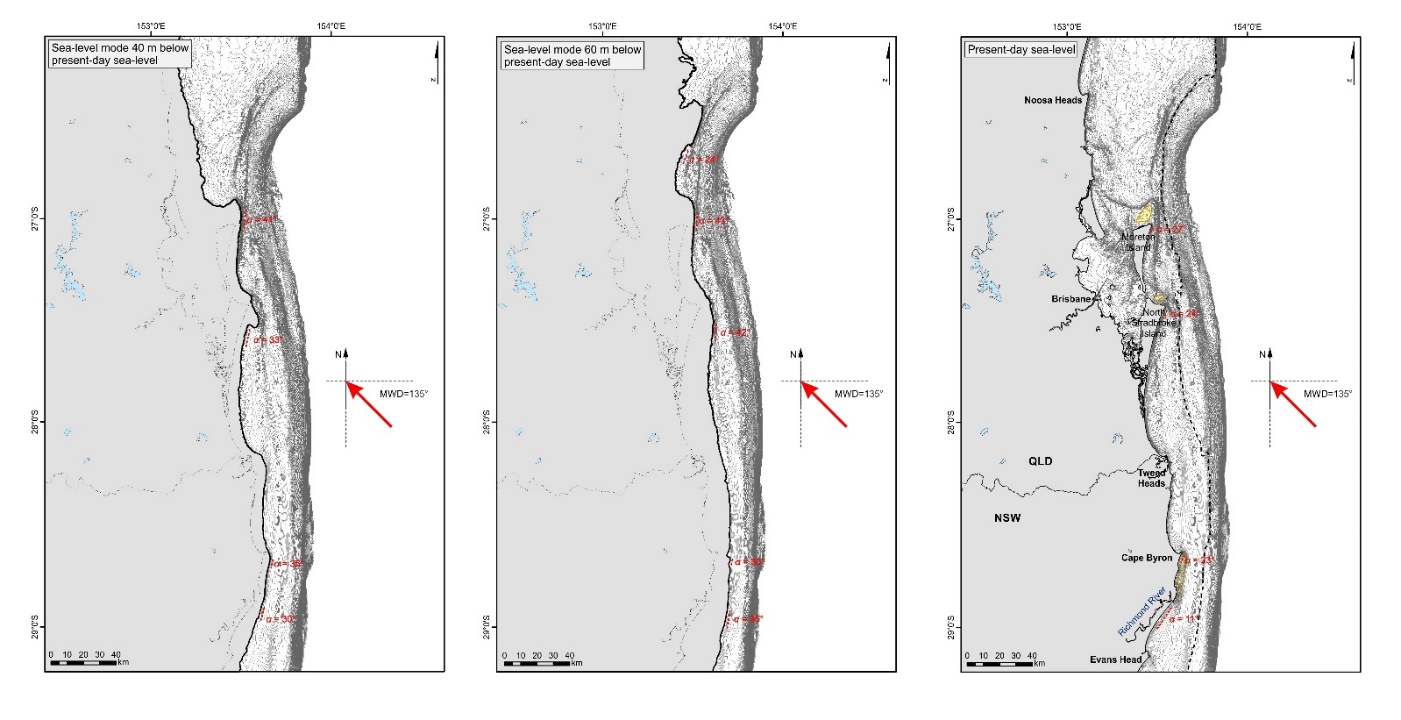


**
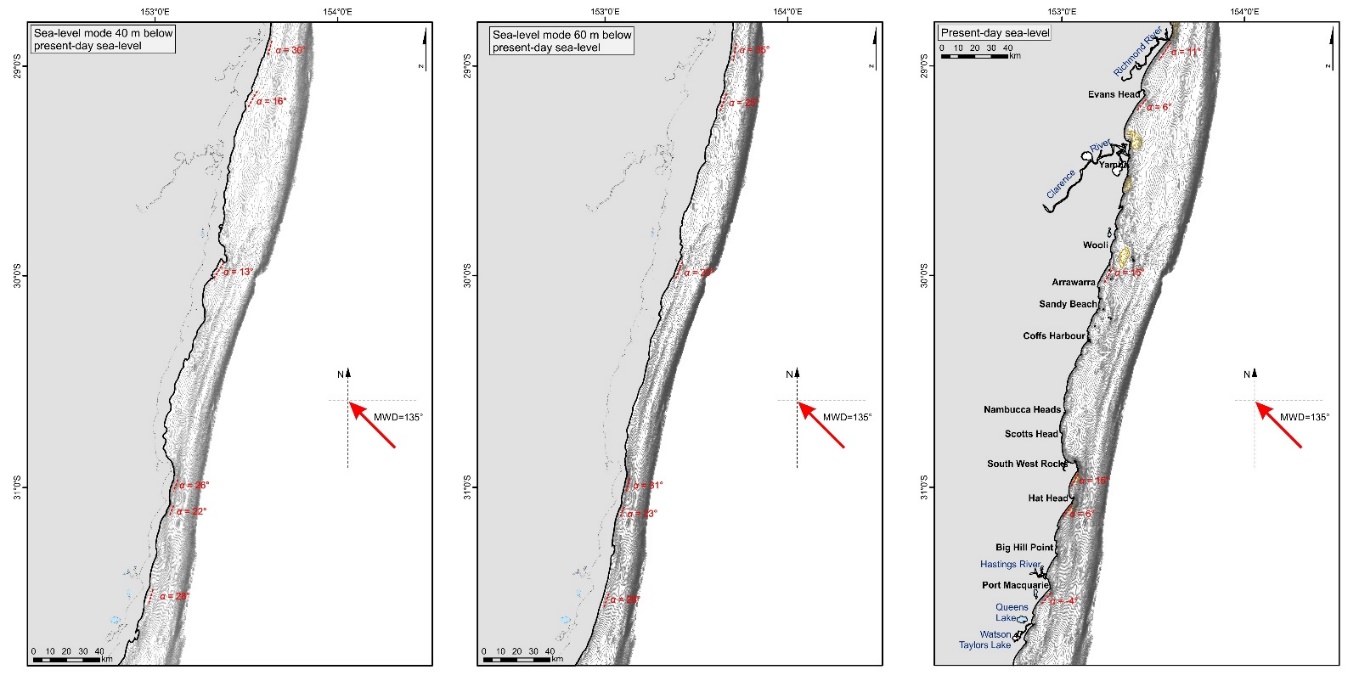
**


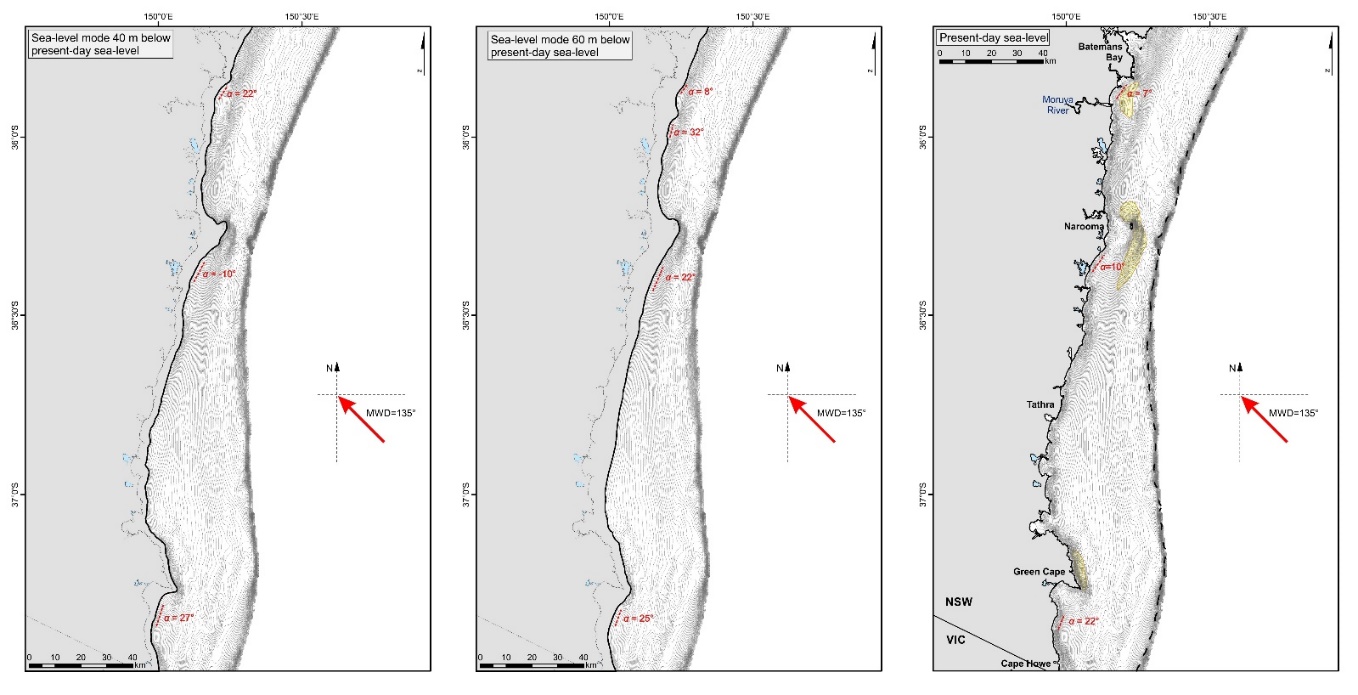


**Figure S3**. Wave obliquity analysis to the 40 m and 60 m sea-level modes paleoshorelines and to present-day shelf and coast, using a mean wave direction (MWD) of 135°. SSBs location are indicated in the present-day shelf configuration (yellow filled areas).

**FIGURE S4**

**
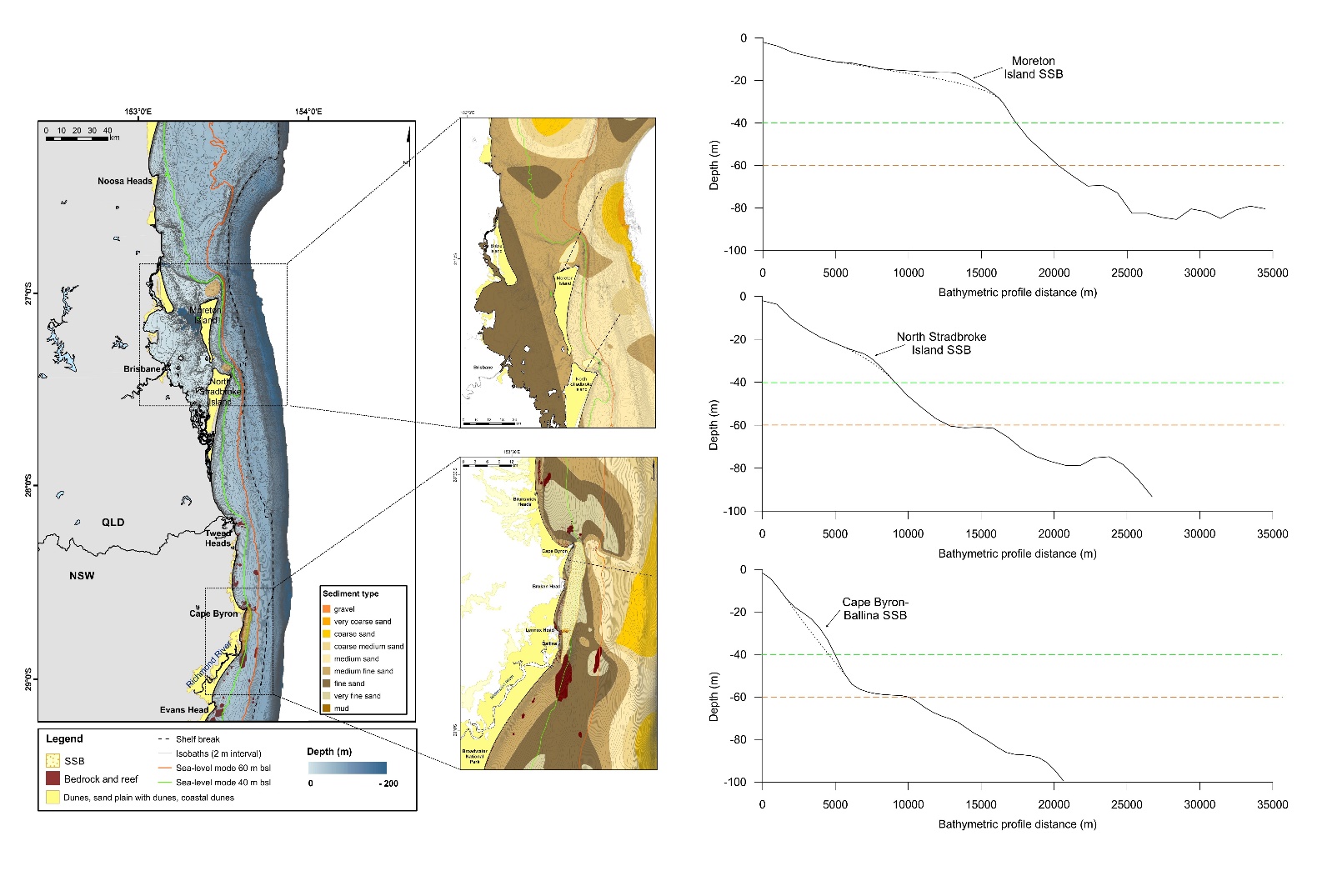
**


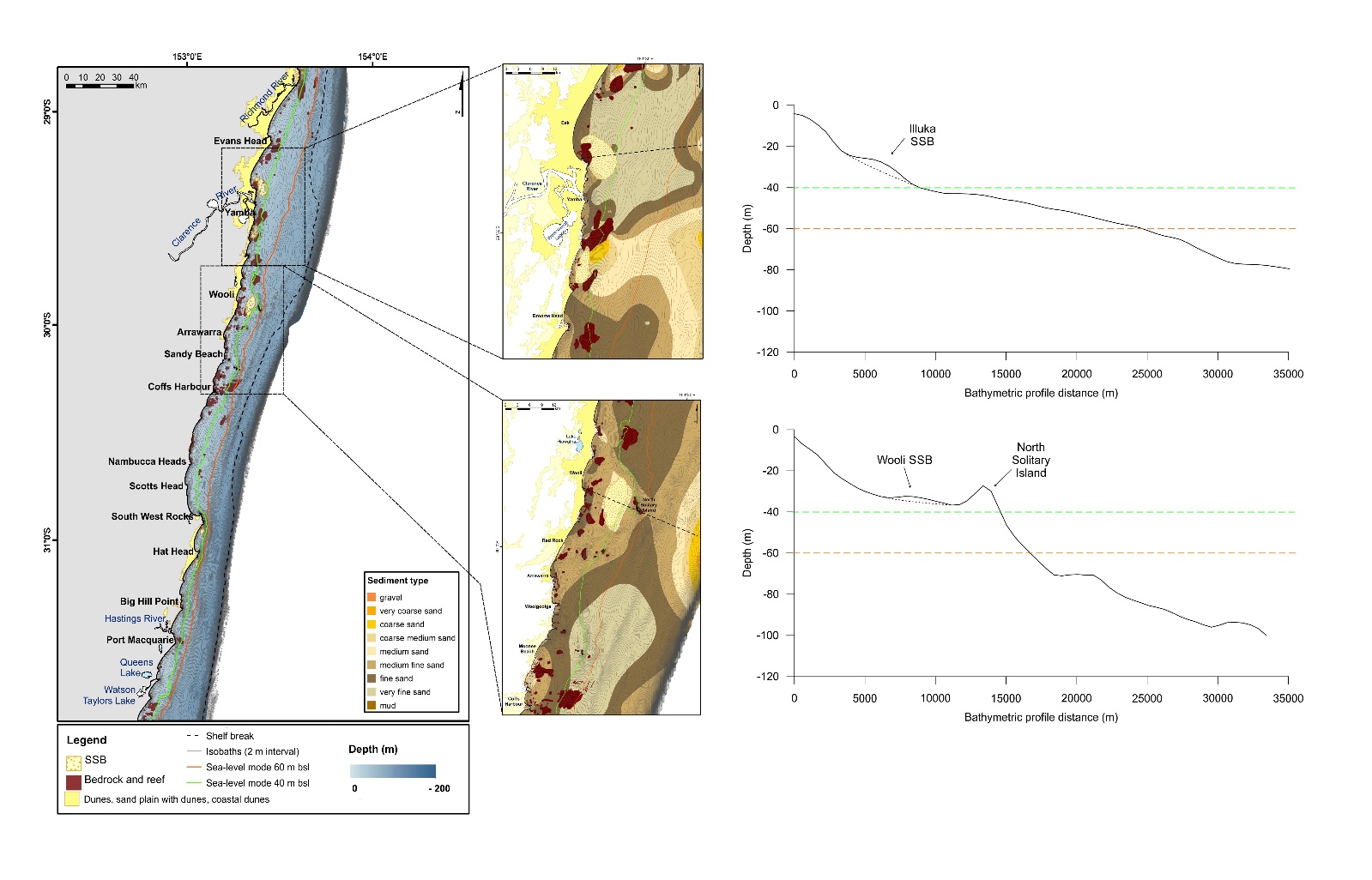


**Figure S4.** Bathymetric composite map of the south-east QLD and north NSW section, including the bedrock, reef and SSBs location and the onshore Quaternary surficial geological units. The 40 m and 60 m isobaths are highlighted in green and orange, respectively, indicating the modal sea-level (i.e., the paleoshorelines location preserved over the present-day south-east Australian shelf). Dashed line squares indicate the zoom-in areas, which include the grain size distribution information and the location of the SSBs. Cross-section bathymetric profiles showed the location of both SSBs, in relation with the modal sea-level depths.

**FIGURE S5**


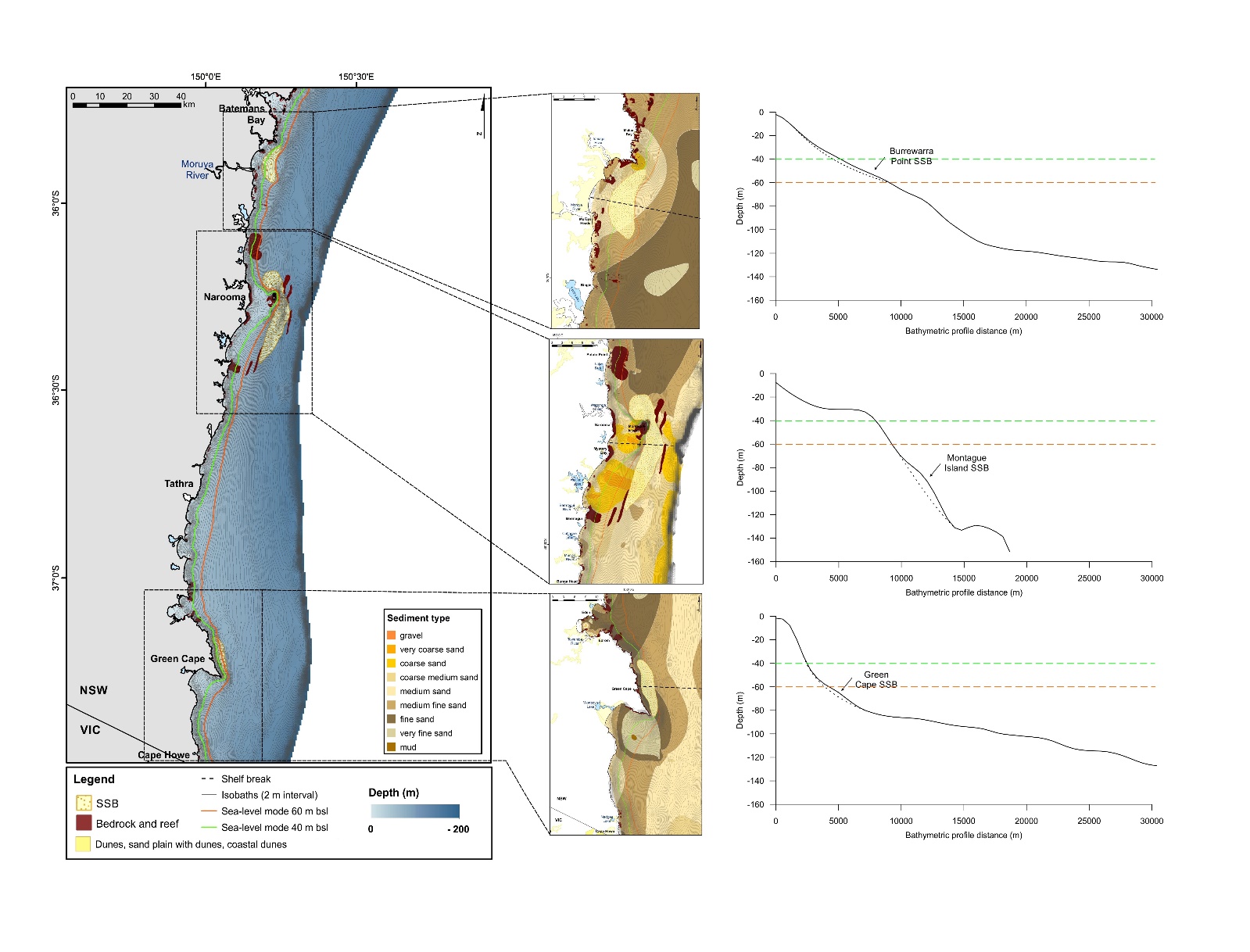


**Figure S5** Bathymetric composite map of the south NSW section, including the bedrock, reef and SSBs location and the onshore Quaternary surficial geological units. The 40 m and 60 m isobaths are highlighted in green and orange, respectively, indicating the modal sea-level (i.e., the paleoshorelines location preserved over the present-day south-east Australian shelf). Dashed line squares indicate the zoom-in areas, which include the grain size distribution information and the location of Burrewarra Point SSB south of Batemans Bay, Montague Island SSB and Green Cape SSB. Cross-section bathymetric profiles showed the location of both SSBs, in relation with the modal sea-level depths.
